# Orthopedic therapeutic surgery for bone metastasis of liver cancer: Clinical efficacy and prognostic factors

**DOI:** 10.3389/fsurg.2022.957674

**Published:** 2022-10-26

**Authors:** Qiujian Lian, Chang Liu, Fengmei Chen, Bingxuan Wang, Miao Wang, Suchi Qiao, Zhengmao Guan, Shuai Jiang, Zhiwei Wang

**Affiliations:** ^1^ Department of Orthopedics, The Third Affiliated Hospital (Eastern Hepatobiliary Surgery Hospital), Naval Medical University (Second Military Medical University), Shanghai, China; ^2^ Department of Orthopedics, The 900th Hospital of Joint Logistic Support Force, Fuzhou, China

**Keywords:** liver neoplasms, bone metastasis, orthopedic surgery, quality of life, prognostic factors, survival

## Abstract

**Objectives:**

In this study, the objectives were to investigate the clinical efficacy of orthopedic therapeutic surgery (OTS) in patients with bone metastasis of liver cancer and explore the prognostic factors.

**Methods:**

The electronic medical records of patients with bone metastasis of liver cancer in the Third Affiliated Hospital of Naval Medical University from September 2016 to August 2021 were retrospectively collected. A total of 53 patients were included. Patients were assigned to the OTS (*n* = 35) or the control group (*n* = 18) based on receiving orthopedic therapeutic surgery or conservative treatment. The pre/posttreatment Karnofsky Performance Status scale (KPS) and numeric rating scale (NRS) scores were compared. Univariate and multivariate Cox regression analyses were used to explore the prognostic factors affecting survival after bone metastasis. Logistic regression analyses were adopted to discover potential factors that contributed to greater KPS score improvement.

**Results:**

The axial bone accounted for 69.8% of all bone metastases. The proportion of multiple bone metastases was 52.8%. After surgery, the median KPS score of the OTS group increased from 60 to 80 (*p *< 0.001), and the median increase in the OTS group was higher than that of the control group (*p *= 0.033). The median NRS score of the OTS group declined from 6 to 2 after surgery (*p *< 0.001), and the median decline in the OTS group was higher (*p *= 0.001). The median survival was 10 months in the OTS group vs. 6 months in the control group (*p* < 0.001). Higher pretreatment KPS scores, undergoing liver primary lesion surgery, and undergoing orthopedic therapeutic surgery were protective factors of survival. Undergoing orthopedic therapeutic surgery greatly improved the KPS score.

**Conclusions:**

Orthopedic therapeutic surgery for bone metastasis of liver cancer provides benefits to the quality of life. Patients who have their primary liver lesions removed, undergo orthopedic therapeutic surgery, and have a better physical condition before treatment tend to have longer survival.

## Introduction

Liver cancer, including hepatocellular carcinoma (HCC), intrahepatic cholangiocarcinoma (ICC), and combined hepatocellular–cholangiocarcinoma (CHC), is one of the most common malignant neoplasms in Asia (1, 2). The most recent 2018 data indicated that the age-standardized incidence rates of liver cancer in China and South Korea were above 15 per 100,000 (1). HCC accounts for more than 90% of liver cancer (2). Previously, the survival time of HCC patients was short and the symptoms of bone metastasis were rarely reported due to the poor control of the primary lesions (3). Recently, with the development of the therapy strategy, the survival time of liver cancer patients has been prolonged. Correspondingly, a higher diagnostic rate of liver cancer bone metastasis attracts more attention. In recent reports, bone has become the second most common metastatic site of HCC, accounting for 25% of extrahepatic metastases of HCC (4–6). The existence of bone metastasis can cause pain, pathological fractures, paralysis, and other skeletal-related events, which seriously affect patients’ quality of life.

Studies have revealed that radiotherapy for bone metastasis of liver cancer brings certain therapeutic benefits (7–11). However, radiotherapy cannot maintain and restore bone stability, which may lead to pathological fractures. In addition, there are risks of radiation resistance and nontarget damage to important adjacent structures (e.g., spinal cord and bone marrow) (12–14). The new concept holds that if there are only limited bone metastases sites, especially for patients whose primary tumor has been radically resected, resection of bone lesions is expected to cure the tumor and improve patients’ survival rate. In this situation, *en bloc* resection and reconstruction of the metastatic sites should be performed following the principles of primary malignant bone tumor surgery (15). For patients with better physical conditions, especially those with a longer expected survival time and limited bone metastases, surgery can eliminate the lesions to the greatest extent and provide immediate bone stability, which may benefit patients more.

To our knowledge, no previous research focused on surgical treatment for bone metastasis of liver cancer. As the main partner hospital of the China National Center for Liver Cancer, the Third Affiliated Hospital of Naval Medical University (Eastern Hepatobiliary Surgery Hospital) has treated a large number of liver cancer patients, many of whom have developed bone metastasis. Herein, we retrospectively analyzed the clinical information of patients with bone metastasis of liver cancer treated in our hospital and explored the potential factors that affect patients’ survival and quality of life.

## Patients and methods

### Patients

This retrospective study was approved by the Ethics Committee of the Third Affiliated Hospital of Naval Medical University (Second Military Medical University). This study was conducted in accordance with the principle of the Helsinki Declaration. Written informed consent to participate in this study was obtained from all patients.

The electronic medical record system of the Third Affiliated Hospital of Naval Medical University (Eastern Hepatobiliary Surgery Hospital) was searched retrospectively. Patients whose primary diagnosis contained the expected keywords (i.e., “malignant tumor,” “metastasis,” “occupying lesion,” “pathological fracture,” and “compression fracture”) were collected. In total, 154 patients were preliminarily selected. Furthermore, we reviewed their medical records and excluded unwanted data according to the inclusion and exclusion criteria. All cases of primary liver cancer were assessed, regardless of the histology and treatment of the primary liver lesion. Eventually, 53 patients were enrolled in this research. The last follow-up date was March 1, 2022. One patient lost to follow-up 6 months after the diagnosis of bone metastasis. Five patients survived at the end of the follow-up.

To eliminate possible biases, we carefully designed the inclusion and exclusion criteria. The inclusion criteria for this study were as follows: (1) Bone metastasis was diagnosed between September 2016 and August 2021; (2) The primary tumor was pathologically diagnosed as liver cancer, or the bone lesion was pathologically confirmed as the origin of liver cancer; (3) Patients received surgical or conservative treatment in our hospital; (4) Patients were assessed as Child–Pugh class A or B when diagnosed with bone metastasis; (5) The expected survival time was more than 3 months after diagnosis of bone metastasis.

The exclusion criteria for this study were as follows: (1) Existence of extra-osseous distant metastasis; (2) The bone lesions received radiotherapy; (3) Responsible bone metastasis lesions that cause symptoms were unresectable; (4) Patients who had other medical conditions that might affect their life expectancy; (5) Patients who had primary neurological disorders that might affect postoperative function; (6) Existence of portal vein tumor embolus.

### Group and treatment choices

For each patient, a variety of imaging examinations, including ultrasound, x-ray, enhanced CT and MRI, and PET/CT, were applied to confirm the sites and number of bone metastasis and to help exclude metastases in other organs. Blood tests such as liver and kidney function, electrolytes, coagulation function, and tumor markers were also routinely used to assist in evaluating the basic condition of patients. The biopsy of bone metastases was not required for all patients.

**Figure 1:**
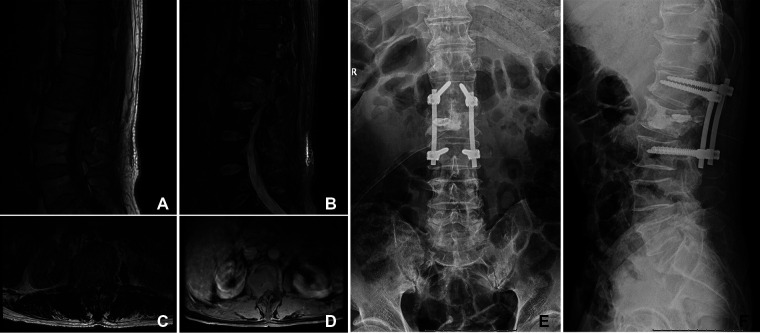
A 66-year-old woman suffered from HCC with multiple metastases of the spine and pelvis. The primary lesion of the liver was not resected. The symptoms were located in the lumbar 2 vertebra. She underwent orthopedic therapeutic surgery. (**A–D**) The L2 vertebral metastases in MRI. (**E,F**) The patient received intralesional resection with internal fixation and PKP. PKP, percutaneous kyphoplasty.

**Figure 2:**
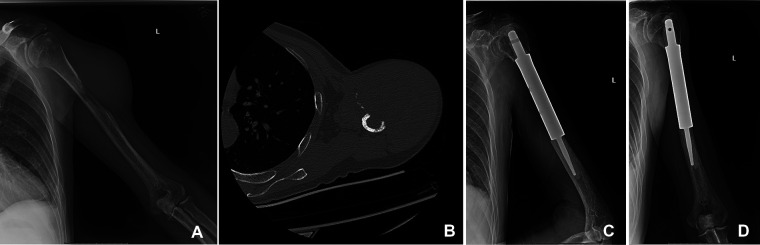
A 50-year-old man suffered from HCC with metastasis of the left humerus. The primary lesion of the liver was resected. (**A,B**) The bone metastasis in the middle part of the left humerus in x-ray and CT. (**C,D**) The patient underwent segmental tumor resection and artificial tumor prosthesis reconstruction.

To explore the different outcomes between orthopedic therapeutic surgery (OTS) and conservative treatment, we divided 53 patients into the OTS group and the control group. Orthopedic therapeutic surgery includes radical and palliative surgery, and excludes diagnostic surgery. The common radical surgery includes artificial tumor prosthesis replacement and *en bloc* resection. The common palliative surgery includes intralesional resection (with or without internal fixation), percutaneous vertebroplasty, percutaneous kyphoplasty, or a combination (Figures 1, 2).

The decision of performing orthopedic therapeutic surgery was made by comprehensively considering the patient's local condition of bone metastases, the degree of pain, the risk of pathological fracture, the physical condition, the life expectancy, and the patient's willingness. The surgery was performed by experienced surgeons. The orthopedic-related conservative (nonsurgical) treatment included physiotherapy, bisphosphonates, and pain-relief medication such as nonsteroidal anti-inflammatory drugs and opioids. For patients who did not meet the surgery criteria or refused surgery, orthopedic conservative treatment was exerted. At the same time, chemotherapy, radiotherapy, targeted therapy, and immunotherapy were performed selectively according to the treatment plan of the Hepatobiliary Department.

Physical therapy was started early after surgery to prevent complications such as venous thrombosis and hypostatic pneumonia. Systematic rehabilitation exercises were carried out in the hospital or at home under the guidance of a doctor.

### The assessment of physical condition and pain level

The Karnofsky Performance Status scale (KPS) score was utilized to evaluate the patient's physical condition and functional impairment. A higher KPS score meant better physical condition and less functional impairment (16). The numeric rating scale (NRS) score was adopted to grade the patient's degree of pain. A lower NRS score meant less pain (17). These two scores were determined before treatment and 1 month after treatment. The changes were scrutinized to measure the improvement in the patient's quality of life.

### Statistical analysis

For the measurement data conforming to the normal distribution, mean ± standard deviation was used, and the Student's *t-*test was applied for comparison. For the measurement data that did not conform to the normal distribution, the median (range) was utilized to display, and the Mann–Whitney *u*-test was applied for comparison. The Chi-square test or Pearson test was performed for comparison of counting data.

The median (range) of pre/posttreatment KPS and NRS were calculated. The pre/posttreatment KPS scores in the OTS group or control group were compared, respectively, using the Mann–Whitney *u*-test, so did the comparison of posttreatment KPS scores between OTS and the control group. The same statistical method was performed in comparison of the NRS scores.

The univariate and multivariate Cox regression was used to explore the potential risk factors of survival time after bone metastasis. Disease-related death during follow-up was defined as the primary outcome. For categorical variables, the Kaplan–Meier method and log-rank test were applied to preliminarily probe risk factors. For continuous variables, the univariate Cox regression analysis was applied to initially explore possible prognostic factors. The variables whose *p*-value was under 0.2 were enrolled in multivariate Cox regression analysis, and a stepwise procedure was executed to correct confounding variables. To note, the variables that had a potential collinear relationship were omitted.

The univariate and multivariate logistic regression was exploited to discover potential influence factors of greater KPS score improvement (i.e., posttreatment KPS score minus pretreatment KPS score was greater than or equal to 20). The greater KPS score improvement was defined as the outcome. The univariate logistic regression analysis was exploited to initially explore possible influence factors. The variables whose *p*-value was under 0.2 were accepted in multivariate logistic regression analysis, and a stepwise procedure was performed to correct confounding variables. The same, the variables that had a potential collinear relationship were ruled out.

All statistical analyses were processed using IBM SPSS Statistics 25.0, and *p *< 0.05 was considered statistically significant.

## Results

### Patients’ clinical characteristics

A total of 53 patients were enrolled in the cohort. Patients’ baseline data were exhibited in Table 1. Among them, 35 patients (66%) underwent orthopedic therapeutic surgery and 18 patients received conservative treatment. Men were the majority in both the OTS group and the control group (74.3% and 83.3%, respectively). The majority of patients were diagnosed with HCC (73.6% in the whole cohort) by the pathological examination, and no one was diagnosed with CHC. For the OTS group, only two patients underwent radical surgery and the remaining 33 patients received palliative surgery. The median follow-up duration was 8 months (range 2–30). The anatomical distribution of bone metastasis was listed in Table 2.

**Table 1:** Clinical characteristics of patients with bone metastasis of liver cancer.

	*n* (%) or mean ± SD or median (range)			*p-*value
	OTS group (*n* = 35)	Control group (*n* = 18)	Total	
Male/female	26 (74.3)/9 (25.7)	15 (83.3)/3 (16.7)	41 (77.4)/12 (22.6)	0.730
Age (years)	60.2 ± 10.2	62.4 ± 10.0	60.9 ± 10.1	0.460
Pathological type				0.191
HCC	28 (80.0)	11 (61.1)	39 (73.6)	
ICC	7 (20.0)	7 (38.9)	14 (26.4)	
Liver primary lesion surgery	27 (77.1)/8 (22.9)	3 (16.7)/15 (83.3)	30 (56.6)/23 (43.4)	<0.001*
Multiple bone metastases	18 (51.4)/17 (48.6)	10 (55.6)/8 (44.4)	28 (52.8)/25 (47.2)	0.776
Sites of bone metastasis				0.328
Axial bones only	26 (74.3)	11 (61.1)	37 (69.8)	
Appendicular bones only	5 (14.3)	2 (11.1)	7 (13.2)	
Mixed	4 (11.4)	5 (27.8)	9 (17.0)	
Pathological fracture	14 (40.0)/21 (60.0)	2 (11.1)/16 (88.9)	16 (30.2)/37 (69.8)	0.030*
Pretreatment KPS score^a^	60 (30–70)	60 (50– 80)	60 (30–80)	0.403
Pretreatment NRS score^a^	6 (4–10)	5 (4–6)	6 (4–10)	<0.001*
AFP positive	13 (37.1)/22 (62.9)	5 (27.8)/13 (72.2)	18 (34.0)/35 (66.0)	0.495
AFP-L3 positive	14 (40.0)/21 (60.0)	4 (22.2)/14 (77.8)	18 (34.0)/35 (66.0)	0.196
PIVKA positive	15 (42.9)//20 (57.1)	6 (33.3)/12 (66.7)	21 (39.6)/32 (60.4)	0.502

OTS, orthopedic therapeutic surgery; HCC, hepatocellular carcinoma; ICC, intrahepatic cholangiocarcinoma; KPS, Karnofsky Performance Status scale; NRS, numeric rating scale; AFP positive, AFP level ≥20 ng/ml; AFP-L3 positive, AFP-L3 percentage ≥10%; PIVKA positive, PIVKA level >40 mAU/ml.

^a^ Expressed as median (range).

* p-value < 0.05.

**Table 2:** Distribution of bone metastasis sites.

Sites of bone metastasis	OTS group	Control group	Total
Axial bones only	26	11	37
Thoracic vertebra	5	2	7
Lumbar vertebra	8	3	11
Sacral vertebra	1	1	2
Rib	1	/	1
Multiple axial bones^a^	11	5	16
Appendicular bones only	5	2	7
Humerus	1	/	1
Radius	1	/	1
Ilium	/	1	1
Femur	1	1	2
Multiple appendicular bones^a^	2	/	2
Mixed^b^	4	5	9

OTS, orthopedic therapeutic surgery.

^a^ Multiple metastases at one anatomical site were categorized as “multiple axial bones” or “multiple appendicular bones” (e.g., one patient with several lumbar metastases was categorized as “multiple axial bones”).

^b^ Multiple metastases occurred in both axial bones and appendicular bones.

The OTS group and the control group were compared in gender, age, pathological type, multiple bone metastases, sites of bone metastasis, pretreatment KPS score, α-fetoprotein (AFP), AFP-L3, and PIVKA. The differences were not statistically significant. It is worth noting that there were statistically significant differences between the two groups in liver primary lesion surgery, pathological fractures, and pretreatment NRS scores. In the OTS group, there was a higher proportion of patients who underwent surgery on the liver primary lesion or suffered pathological fractures, and a higher pretreatment NRS score. These results suggested that these factors may increase the willingness of patients to receive orthopedic therapeutic surgery.

### The benefit of orthopedic therapeutic surgery

The KPS and NRS scores before and one month after treatment were evaluated respectively. The scores pre/posttreatment within or between groups were compared to investigate whether orthopedic therapeutic surgery had an association with better quality of life and pain relief.

**Figure 3:**
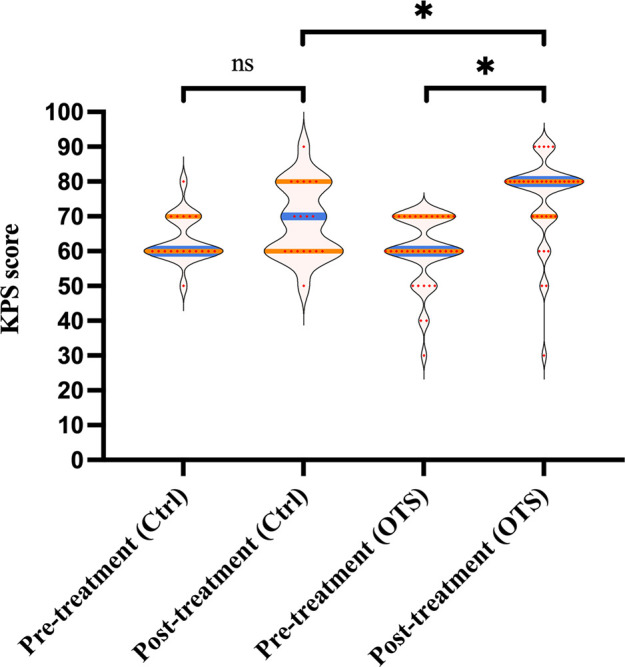
The KPS score pre/posttreatment.

As shown in Figure 3, although the median posttreatment KPS score was higher than the median pretreatment KPS score in the control group (70 vs. 60), it was not statistically significant (*p* = 0.104). The median KPS score of the OTS group increased from 60 (range 30–70) before OTS to 80 (range 30–90) after OTS (*p *< 0.001). Additionally, the median increase in posttreatment KPS score of the OTS group was higher than that of the control group (20 vs. 10), which was statistically significant (*p *= 0.033).

**Figure 4:**
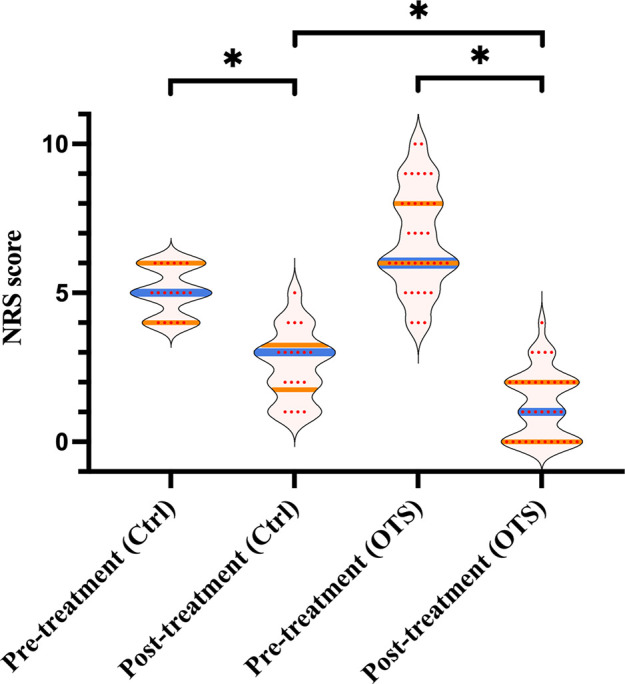
The NRS score pre/posttreatment.

As Figure 4 exhibited, the median NRS score of the OTS group declined from 6 (range 4–10) pretreatment to 2 (range 0–4) one month after treatment (*p *< 0.001). Comparatively, the median NRS score of the control group declined from 5 (range 4–6) before treatment to 3 (range 1–5) after treatment (*p *< 0.001). Moreover, the median decline in posttreatment NRS score of the OTS group was more than that of the control group (4 vs. 2), which was also statistically significant (*p *= 0.001).

These data indicated that orthopedic therapeutic surgery improved postoperative functional status and achieved greater pain relief in patients compared with conservative treatment.

### The impact of orthopedic therapeutic surgery on survival time

**Figure 5:**
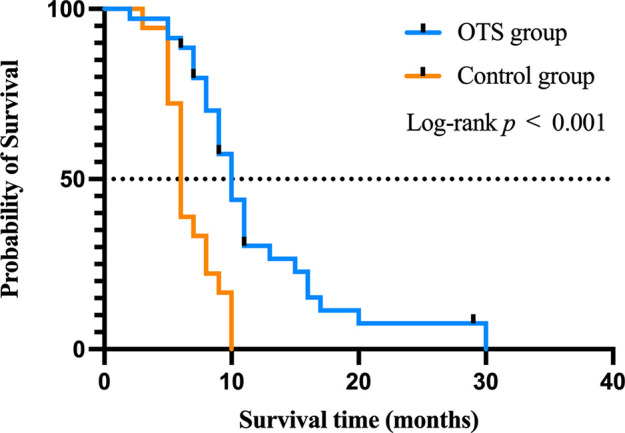
The impact of orthopedic therapeutic surgery and conservative treatment on the survival of patients after bone metastasis.

To further explore the prognostic factors of survival time after bone metastasis, the Cox regression analysis was performed. The median survival time of all patients was 9 months (range 2–30). The survival curves of the OTS group and the control group were drawn by the Kaplan–Meier method (Figure 5). The median survival time of the patients of the OTS group was 10 months (range 2–30) and that of the control group was 6 months (range 3–10). The difference was statistically significant (*p *< 0.001). These results were further investigated in subsequent multivariate Cox regression analysis.

### The prognostic factors of survival time of patients with bone metastasis of liver cancer

To explore the prognostic factors affecting the survival time of patients with bone metastasis of liver cancer and correct for confounding factors, a Cox regression analysis of the clinical data of all patients (*n* = 53) was performed (Table 3).

**Table 3:** Cox regression analysis to identify the prognostic factors of survival time.

Variables	Univariate analysis	Multivariate analysis	
	*p-*value	HR (95% CI)	*p-*value
Sex	0.796		
Age	0.221		
Pathological type^b^ (HCC = 1, ICC = 2)	0.155	—	—
Liver primary lesion surgery^b^	<0.001*	0.243 (0.110–0.540)	0.001*
Multiple bone metastases^b^	0.081	—	—
Number of bone metastasis^a^	0.009*		
Sites of bone metastasis^a^	0.024*		
Pathological fracture	0.784		
Orthopedic therapeutic surgery^b^	<0.001*	0.135 (0.145–0.687)	0.004*
Pretreatment KPS score^b^	0.007*	0.917 (0.879–0.956)	<0.001*
Pretreatment NRS score	0.555		
AFP positive^b^	0.164	—	—
AFP-L3 positive	0.230		
PIVKA positive	0.791		

HR, hazard ratio; CI, confidence interval; HCC, hepatocellular carcinoma; ICC, intrahepatic cholangiocarcinoma; KPS, Karnofsky Performance Status scale; NRS, numeric rating scale; AFP positive, AFP level ≥20 ng/ml; AFP-L3 positive, AFP-L3 percentage ≥10%; PIVKA positive, PIVKA level >40 mAU/ml.

^a^ Due to collinearity with “Multiple bone metastases,” it was not included in the multivariate Cox regression analysis.

^b^ Included in the multivariate Cox regression analysis.

* p-value < 0.05.

The univariate Cox analysis showed that liver primary lesion surgery, orthopedic therapeutic surgery, number of bone metastasis, sites of bone metastasis, and pretreatment KPS score had statistical significance on the survival time after bone metastasis.

Pathological type, liver primary lesion surgery, multiple bone metastases, orthopedic therapeutic surgery, pretreatment KPS score, and AFP positive were further enrolled in the multivariate Cox analysis to correct for confounding factors. (Number of bone metastasis and sites of bone metastasis was excluded due to the collinearity with “Multiple bone metastases”). The final result indicated that a higher pretreatment KPS score, undergoing liver primary lesion surgery, and undergoing orthopedic therapeutic surgery were protective factors, and the differences were statistically significant.

### The influence factors of greater KPS score improvement in patients with bone metastasis of liver cancer

Since the basic physical conditions of advanced cancer patients were important (evaluated by KPS scores), we further explored the potential influence factors of greater KPS score improvement (Table 4).

**Table 4:** The logistic regression to identify the influence factors of greater KPS score improvement.

Variables	Univariate analysis	Multivariate analysis	
	*p-*value	OR (95% CI)	*p-*value
Sex	0.749		
Age	0.668		
Pathological type (HCC = 1, ICC = 2)	0.410		
Liver primary lesion surgery^b^	0.003*	—	—
Number of bone metastasis^b^	0.099	—	—
Sites of bone metastasis^a^	0.134		
Pathological fracture	0.553		
Orthopedic therapeutic surgery^b^	0.009*	8.718 (2.214–35.783)	0.003*
Pretreatment KPS score	0.818		
Pretreatment NRS score	0.319		
AFP positive^b^	0.095	—	—
AFP-L3 positive	0.283		
PIVKA positive	0.592		

Greater KPS score improvement (posttreatment KPS score minus pretreatment KPS score was greater than or equal to 20) was defined as the outcome.

OR, odds ratio; CI, confidence interval; HCC, hepatocellular carcinoma; ICC, intrahepatic cholangiocarcinoma; KPS, Karnofsky Performance Status scale; NRS, Numeric rating scale; AFP positive, AFP level ≥20 ng/ml; AFP-L3 positive, AFP-L3 percentage ≥10%; PIVKA positive, PIVKA level >40 mAU/ml.

^a^ Due to collinearity with “Number of bone metastasis”, it was not included in the multivariate logistic regression analysis.

^b^ Included in the multivariate logistic regression analysis.

* p-value < 0.05.

The univariate logistic regression analysis showed that liver primary lesion surgery and orthopedic therapeutic surgery had statistical significance on the greater KPS score improvement.

Liver primary lesion surgery, orthopedic therapeutic surgery, number of bone metastasis, and AFP positive were further enrolled in the multivariate logistic analysis to correct for confounding factors (“Sites of bone metastasis” was excluded due to the collinearity with “Number of bone metastasis”). The final result indicated that undergoing orthopedic therapeutic surgery was the positive factor that contributed to a greater KPS score improvement and was statistically significant (*p *= 0.003).

## Discussion

Liver cancer is the second most common cause of cancer mortality in the Asia-Pacific region (2), and the incidence of liver cancer is much higher in Asia than in Europe and the Americas, with HBV and other infections being the main risk factors (1, 2, 18). As mentioned above, attributing to advances in diagnosis and treatment, more bone metastases are diagnosed and concerned.

In the study by Si et al., bone metastasis occurred in 9.8% of all HCC patients (34/347) (19). To note, bone metastasis may occur even after the radical resection of the primary tumor (20, 21). The spine is reported to be the most common site of bone metastasis, and about 70% of patients with bone metastases are multiple (6). Previous literature has explored the efficacy of radiotherapy on bone metastasis of liver cancer (8–11). However, for patients with a longer expected survival time, or those whose primary lesions are controlled while the symptoms of bone metastasis are severe, surgery has multiple advantages such as reducing tumor burden, maintaining bone stability, and preventing long-term bone-related complications. Taking advantage of the great number of liver cancer patients in our hospital, this study probed the impact of surgical treatment for bone metastasis of liver cancer and potential prognostic factors of survival time after bone metastasis. As far as we know, there is no previous literature on surgical treatment for bone metastasis of liver cancer.

In this study, the clinical characteristics of all patients were analyzed. In the current cohort, there were more men than women and HCC was predominant, which is consistent with epidemiology (1, 2). Several variables were found to be statistically significant between the OTS and control groups, including liver primary lesion surgery, pathological fractures, and pretreatment NRS score. We infer that patients with these factors were more willing to undergo orthopedic therapeutic surgery for bone metastasis. For patients whose primary liver tumor has been resected, if symptomatic bone metastases have a chance of being resolved, they would more actively seek help from an orthopedic surgeon for better survival. Patients with pathological fractures, or patients with severe pain, on the premise of a long-expected survival time, would be more willing to relieve pain and recover function through orthopedic therapeutic surgery.

We further confirmed that orthopedic therapeutic surgery for bone metastasis improved the quality of life and prolonged the survival time. Univariate and multivariate regression analyses also verified the positive effect of orthopedic therapeutic surgery on bone metastasis in prolonging the survival time and improving KPS scores after bone metastasis. In addition, patients in this study whose primary liver lesions were resected and who were in relatively good physical and functional status pretreatment may have longer survival.

We revealed that active intervention on bone metastasis might help improve the quality of life, which is consistent with some known literature (3, 4). However, it is worth mentioning that liver cancer is not a malignant tumor that can obtain a longer survival period by aggressive surgical treatment of bone metastasis in the existing literature (22, 23). The current results, in which orthopedic therapeutic surgery showed a positive effect on bone metastasis of liver cancer, may result from a combination of multiple factors, including but not limited to the following: (1) Orthopedic surgery reduced the patient's tumor burden and pain as well as improved the patient's physical condition; (2) In the OTS group, a higher primary tumor resection rate might contribute to a longer survival time in conjunction with orthopedic surgery; (3) We have realized in medical practice that patients who were willing to undergo surgery tend to have good economic conditions and have more opportunities to get better treatment plans.

Considering the limited number of cases, only some of the most representative clinical variables were selected for analysis to meet statistical requirements. In some literature studies, KPS score and surgical treatment of primary lesions are considered to have prognostic significance in HCC patients (6, 9, 11, 24), and these factors were reconfirmed by the current study. Some other variables, such as poor liver function, the presence of ascites, and the presence of metastasis in extra-osseous organs, are also considered risk factors for the survival of patients with bone metastasis of liver cancer (3, 6, 9, 11). We did not include these variables because patients with these characters usually had no indication for surgery.

To note, it is important to follow the indications for surgery when performing surgery on bone metastasis. The patients included in the OTS group were carefully evaluated, and those with surgical contraindications were ruled out. In fact, in our clinical practice, some patients underwent surgery out of a strong desire despite contraindications to surgery (not included in the cohort). Unfortunately, several died due to respiratory failure, liver failure, and other reasons within 1 month post surgery, which went against the original intention of surgical treatment for bone metastasis. Before bone metastasis surgery, surgeons should carefully evaluate the indications and contraindications, clarify the pros and cons for the patient, formulate an individualized treatment plan according to the patient's condition, and fully inform the patient and his family (15, 25).

Although this research provided promising results, it still had the following limitations: (1) The number of patients finally included in the cohort was limited due to the low overall incidence of bone metastasis. To meet the statistical requirement (e.g., sample size/variable size ratio), we only selected limited indicators to evaluate related factors in regression analyses. (2) Also, due to the limited sample size, we did not classify and discuss the details of some treatments (e.g., surgical types for bone lesions). In fact, surgical cure of metastatic disease is generally not achievable attributable to the presence of underlying lesions that cannot be detected by current examination methods and the persistent colonization of bone by circulating tumor cells (25, 26). (3) As a retrospective study, we had some inevitable bias. To reduce recall bias and nonresponse bias caused by patients failing to follow-up visits, we conducted a follow-up telephone call 3–5 days after the estimated visit date.

In order to further explore the significance of surgery and the prognostic factors for bone metastasis with liver cancer and reduce potential bias, large cohort, multicenter, randomized, and prospective studies are needed.

## Conclusion

In conclusion, this study indicates that for patients with bone metastasis of liver cancer who meet certain conditions, orthopedic therapeutic surgery can help improve the quality of life and prolong the survival time. Patients with bone metastasis of liver cancer who have their primary liver lesions resected, undergo orthopedic therapeutic surgery, and have a better physical condition before treatment may have a better prognosis.

## Data Availability

The raw data supporting the conclusions of this article will be made available by the authors, without undue reservation.
